# Human Faces Are Slower than Chimpanzee Faces

**DOI:** 10.1371/journal.pone.0110523

**Published:** 2014-10-22

**Authors:** Anne M. Burrows, Lisa A. Parr, Emily L. Durham, Lea C. Matthews, Timothy D. Smith

**Affiliations:** 1 Department of Physical Therapy, Duquesne University, Pittsburgh, Pennsylvania, United States of America; 2 Department of Anthropology, University of Pittsburgh, Pittsburgh, Pennsylvania, United States of America; 3 Department of Psychiatry and Behavioral Science, Center for Translational Neuroscience, Yerkes National Primate Research Center, Emory University, Atlanta, Georgia, United States of America; 4 Department of Health Management Systems, Duquesne University, Pittsburgh, Pennsylvania, United States of America; 5 School of Physical Therapy, Slippery Rock University, Slippery Rock, Pennsylvania, United States of America; University of Goettingen, Germany

## Abstract

**Background:**

While humans (like other primates) communicate with facial expressions, the evolution of speech added a new function to the facial muscles (facial expression muscles). The evolution of speech required the development of a coordinated action between visual (movement of the lips) and auditory signals in a rhythmic fashion to produce “visemes” (visual movements of the lips that correspond to specific sounds). Visemes depend upon facial muscles to regulate shape of the lips, which themselves act as speech articulators. This movement necessitates a more controlled, sustained muscle contraction than that produced during spontaneous facial expressions which occur rapidly and last only a short period of time. Recently, it was found that human tongue musculature contains a higher proportion of slow-twitch myosin fibers than in rhesus macaques, which is related to the slower, more controlled movements of the human tongue in the production of speech. Are there similar unique, evolutionary physiologic biases found in human facial musculature related to the evolution of speech?

**Methodology/Prinicipal Findings:**

Using myosin immunohistochemistry, we tested the hypothesis that human facial musculature has a higher percentage of slow-twitch myosin fibers relative to chimpanzees (*Pan troglodytes*) and rhesus macaques (*Macaca mulatta*). We sampled the orbicularis oris and zygomaticus major muscles from three cadavers of each species and compared proportions of fiber-types. Results confirmed our hypothesis: humans had the highest proportion of slow-twitch myosin fibers while chimpanzees had the highest proportion of fast-twitch fibers.

**Conclusions/significance:**

These findings demonstrate that the human face is slower than that of rhesus macaques and our closest living relative, the chimpanzee. They also support the assertion that human facial musculature and speech co-evolved. Further, these results suggest a unique set of evolutionary selective pressures on human facial musculature to slow down while the function of this muscle group diverged from that of other primates.

## Introduction

Movement of the lips is controlled by the facial musculature (or facial expression musculature), present in all vertebrates [1]. Among skeletal musculature, facial muscles are unique because they attach into the skin of the face and scalp, controlling size and shape of the openings for the eyes, external nares, and mouth. In mammals, this musculature takes on an additional function as a means to produce visual communication signals, deforming the facial mask into expressions of emotion [1]. Primates have a phylogenetically conserved arrangement of facial muscles but there is variation within the order in the complexity of expressions and displays produced. This variation among primate species seems to be largely dependent upon factors such as group size, time of day activity, and environment [1].

Most primates use visual signals as part of their social communication repertoire [1], [2]. Part of the visual signal repertoire includes facial expressions of emotion and many primate species have well-documented, complex repertoires of facial displays/facial expressions [3]–[7]. In addition many primate species use auditory pathways as a means of communication. As primates, humans also use facial displays/expressions as part of our visual signal repertoire but human social communication is achieved primarily through a coarticulation of visual (facial displays/expressions) and auditory pathways with the unique evolutionary innovation of speech [8]–[10]. Speech is distinguished from other types of primate vocalizations in its combination of bimodally rhythmic, acoustic output plus visual output [8], [11]–[13] which depends upon contraction of facial muscles to move the lips. Movements of the lips work together in a highly correlated mechanism with the sounds produced during speech to generate “visemes”, visual phonemes that aid in the perception of speech [9], [14]. Disruption of this correlated acoustic and visual behavior alters production of visemes and reduces speech intelligibility, a phenomenon described by the McGurk Effect [8], [9]. Further, the lips themselves act as articulators in human speech as part of the supralaryngeal portion of the vocal tract [10]. Clearly, precise and highly regulated movement of the lips is an integral part of human speech. Mounting comparative evidence focusing on the evolution of speech suggests that it may have evolved from the rhythmic facial expressions of ancestral primates, such as the lip-smacking gesture in rhesus macaques, geladas, and chimpanzees [15], [16].

Spontaneous facial expressions of emotion in primates (including humans) occur rapidly and last only a short period of time [17]–[20]. However, movements of the face/lips related to the production of speech require a coordinated, rhythmic movement of the facial features with a more controlled, sustained muscle contraction, especially in muscles that attach into the lips [11]–[15].

A recent study found that musculature of the human tongue contains a higher proportion of slow-twitch myosin fibers than found in rhesus macaques, which is related to the slower, more controlled movements of the human tongue used in the production of speech [21]. Are there similar physiological biases found in human facial musculature related to the evolutionary innovation of speech? The present study tests the hypothesis that humans have a significantly higher percentage of slow-twitch vs. fast-twitch myosin fibers in facial muscles attaching into the lips relative to rhesus macaques (*Macaca mulatta*) and chimpanzees (*Pan troglodytes*). Chimpanzees are our closest living relative, are often used in models of human evolution, and have a well-documented facial display repertoire [5]. Rhesus macaques are frequently used as a model of human social behavior and cognition [4]. They live in complex multimale-multifemale social groups and have an intricate repertoire of facial expressions including formal signals [22]. However, chimpanzees are more closely related to humans and also possess a large repertoire of facial expressions and gestures [5], [23]. To determine whether muscle fibers in humans evolved in conjunction with speech, comparisons with chimpanzees are necessary.

## Materials and Methods

This study used myosin immunohistochemistry across a phylogenetic range of primate taxa to test our hypothesis. Sections from the upper lip (containing the orbicularis oris muscle [OOM]) and from the zygomaticus major (ZM) muscle with overlying skin were sampled from humans (N = 3), rhesus macaques (N = 3) and chimpanzees (N = 3). These muscles were chosen because they are involved in both facial displays and in vocalizations/speech in all study groups and attach into the lips. The human specimens were gathered from cadavers at Duquesne University and Slippery Rock University Gross Anatomy Labs; rhesus macaque and chimpanzee specimens were obtained from Yerkes National Primate Research Center after the animals died from natural causes. All samples were fixed with either formaldehyde (human) or 10% buffered formalin (macaque and chimpanzee).

Samples were prepared for paraffin based histology following methodology from Burrows et al. [24] and Muchlinski et al. [25]. Each muscle was sectioned at 6–10 µm thickness. From each muscle in each specimen, 150 to 300 sections that were spaced five sections apart were mounted on slides. This methodology generated sections that were representative of the entire muscle. Future studies will be aimed at differentiating the isoforms of fast- twitch (type II) myosin fibers but the present study only focused on differentiating slow-twitch fibers (type I) vs. fast-twitch (type II) myosin fibers. Mouse monoclonal antibodies were used as primary antibodies to slow myosin (ab11083, Clone NOQ7.5.4D, Abcam, Inc.) and fast myosin (ab7784, Clone MY-32, Abcam, Inc.). A random selection of three to five slides per individual containing three to four muscle sections per slide were chosen for immunohistochemistry using each primary antibody which yielded 18–40 sections for each muscle in each of the study groups.

To prepare tissues for immunohistochemistry, de-paraffinized rehydrated sections were subjected to enzymatic retrieval with 0.5% trypsin for slow myosin, or an overnight epitope retrieval with Tris-EDTA buffer for fast myosin staining. Endogenous peroxidase activity was blocked by 0.9% hydrogen peroxide in methanol and sections were pretreated with 5% normal goat serum. The primary antibodies to slow myosin (1:2000) and fast myosin (1:1500) were diluted in 5% normal goat serum and were incubated overnight at 4°C. After this, sections were washed with PBS and biotinylated goat anti-mouse antibody diluted 1:200 in 5% normal goat serum was applied. Sections were then again washed and incubated with Vectastain ABC reagent (Vector Laboratories). Finally, sections were exposed to 3,3′-diaminobenzidine diaminobenzidine tetrahydrochloride (DAB) (Vector Laboratories) for two minutes, the reaction was stopped with water, and the sections were dehydrated, cleared, and mounted with permount (Fisher Scientific).

### Determination of fiber type proportions

**Figure 1:**
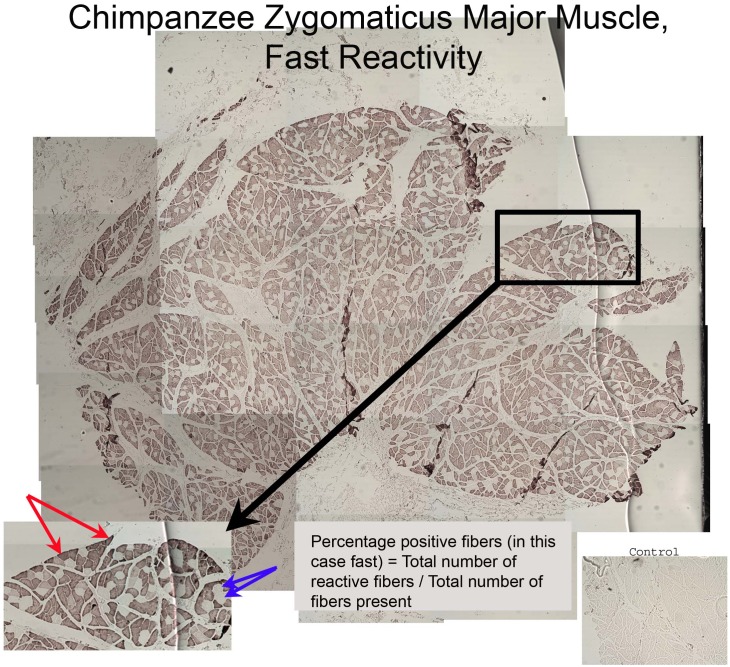
Methodology for calculating percentage of reactive fibers. This is a cross-section through the zygomaticus major muscle of one chimpanzee specimen that was stained for fast-myosin reactivity. Percentages of reactive fibers were determined by counting all of the reactive fibers in each composite image (here, the reactive fibers are stained brown) then dividing that count by the total number of fibers present in the composite image. Blue arrows are pointing to examples of fibers showing positive reactivity for fast-myosin staining; red arrows are pointing to examples of fibers that were non-reactive.

The proportions of fiber types were determined by selecting 3–10 sections stained for identification of each fiber type for each individual in both the OOM and ZM muscles and photographing the entire cross section of each muscle [20]. We created composite images of muscle sections and assessed fiber type proportions using ImageJ (NIH). In order to derive percentages of fast and slow fiber types, all of the reactive fibers in each composite were counted, then divided by the total number of fibers present. The resulting percentage of reactive fibers in each composite was used to calculate the mean percentage of fiber type present in each muscle for each species (see Figure 1).

Because the resulting percentages of myosin fibers were not normally distributed each percentage was arcsine-transformed [26] and these transformed values were compared among groups using one-way ANOVAs in SPSS (v. 20). Where significant (p<0.05) group-wide mean differences existed, a *post-hoc* Least Squares Difference test was employed between groups to locate between-group significant differences.

### Ethics statement

All animal tissue used in the present study was derived from cadavers that died of natural causes at Yerkes National Primate Research Center. Because the animals were not part of a study at Duquesne University nor were they killed as part of any study, the Duquesne University IACUC did not review any proposals concerning the present study. All human tissue used in the present study was derived from cadavers used in human gross anatomy courses at Duquesne University and Slippery Rock University. Because these individuals were dead, no written or verbal consent was obtained from them prior to their inclusion in the present study. The IRB of both Duquesne University and Slippery Rock University do not review proposals dealing with human tissues derived from cadavers used in human gross anatomy courses nor do they require consent from them. However, the Human Gifts Registry that received and distributed these cadavers approved the use of the samples. When individuals decide to will their bodies to science, the appropriate Human Gifts Registry has no mechanism for promising that the individuals’ remains will be used either for gross anatomy course dissections or for research purposes. Individuals who will their bodies to science are notified that either or both of these scientific activities may occur with their remains. Only individuals who have requested that their remains be donated to science can enter the Human Gifts Registry and it is this registry that distributes cadavers. All identifying information about the individual cadavers used in the present study is held by the gross anatomy course directors or department chairs of Duquesne University and Slippery Rock University but was not accessed for the purposes of this study.

## Results and Discussion

**Figure 2:**
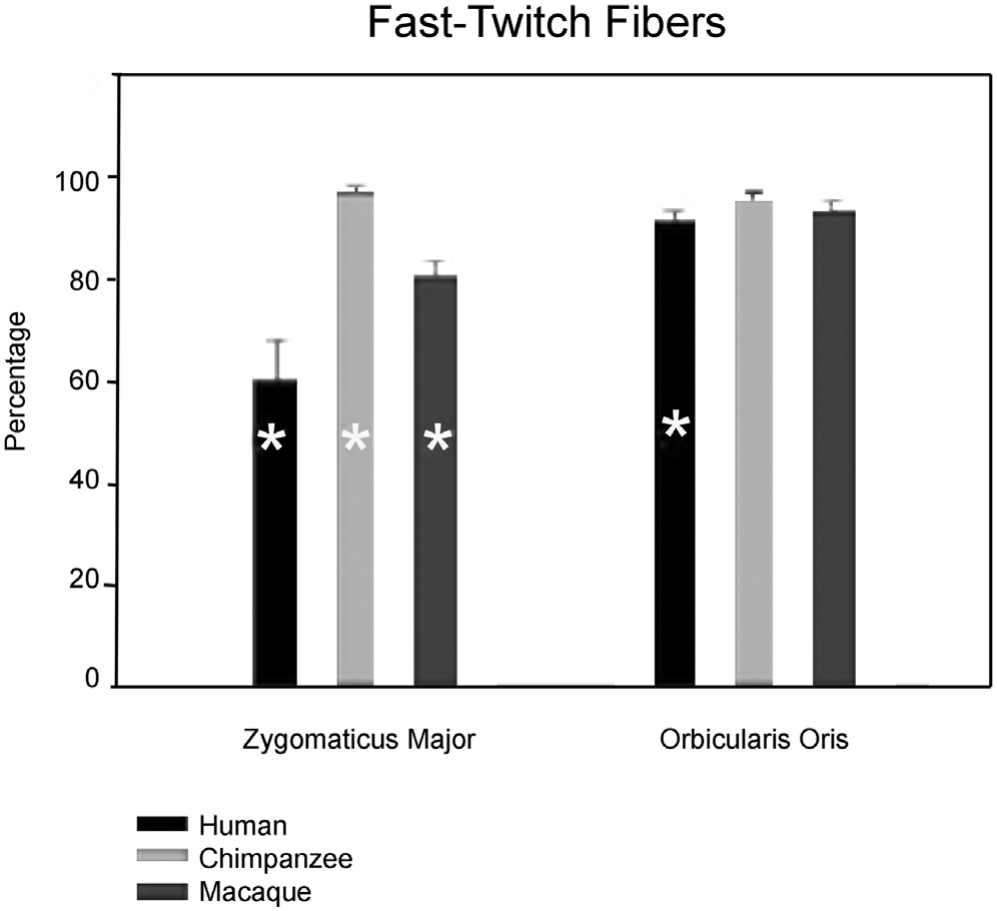
Percentages of fast-twitch myosin fibers in facial muscles. Results of one-way ANOVA and *post-hoc* Least Squares Difference testing in the zygomaticus major muscle indicate that chimpanzees had the highest percentage of fast-twitch myosin fibers and humans had the lowest (chimpanzee > macaque > human) at the p<0.05 level of statistical significance. In the orbicularis oris muscle, humans had the lowest percentage of fast-twitch fibers (chimpanzee = macaque > human) at the p<0.05 level of statistical significance. The presence of a “*” indicates that the group mean percentage was significantly different from the other groups. Means with SEM are shown.

**Figure 3:**
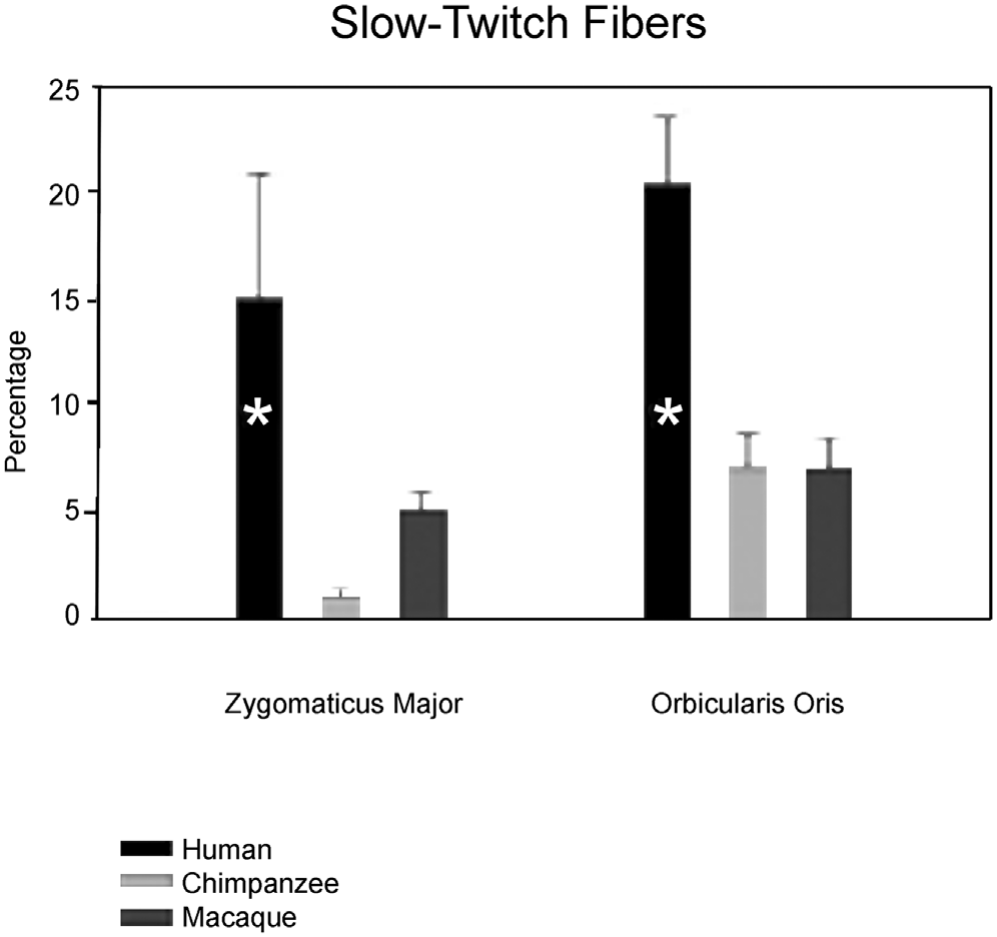
Percentages of slow-twitch myosin fibers in facial muscles. Results of one-way ANOVA and *post-hoc* Least Squares Difference testing in the orbicularis oris muscle indicate that humans have the highest percentage of slow-twitch myosin fibers (human > chimpanzee = macaque) at the p<0.05 level of statistical significance. For the ZM, Least Squares Difference testing was unable to separate chimpanzees from humans at the p<0.05 level of statistical significance. The presence of a “*” indicates that the group mean percentage was significantly different from the other groups. Means with SEM are shown.

Figures 2 and 3 depict results of one-way ANOVA among the three study groups for relative percentages of myosin fibers. Tables S1 and S2 show the mean raw percentages among the groups as well as the mean arcsine-transformed percentages. Results of one-way ANOVA revealed significant (p<0.05) mean intergroup differences for both fast-twitch and slow-twitch myosin percentages in the zygomaticus major muscle (ZM) and the orbicularis oris muscle (OOM). Chimpanzees had the highest proportion of fast-twitch fibers in both muscles (97% in the ZM and 96% in the OOM) followed by rhesus macaques (80.5% in ZM and 93% in OOM). Humans had the lowest percentage of fast-twitch fibers in both muscles (60% in ZM and 91% in OOM). These differences were statistically (p<0.05) significant for both the ZM (df = 2, p = 0.03) and the OOM (df = 2, p = 0.01).

Results of *post-hoc* Least Squares Difference testing revealed that all three groups were significantly different from one another in both muscles. In the ZM, the chimpanzee had the greatest percentage of fast-twitch fibers followed by rhesus macaques (chimpanzee > rhesus macaque > human; chimpanzee v. human p = 0.00; chimpanzee v. rhesus macaque p = 0.02; rhesus macaque v. human p = 0.02). In the OOM, both chimpanzees and rhesus macaques had the highest percentage of fast-twitch fibers (chimpanzee = rhesus macaque > human) but results of *post-hoc* Least Squares Difference testing failed to separate rhesus macaques from humans (chimpanzee v. human p = 0.04; chimpanzee v. rhesus macaque p = 0.33; rhesus macaque v. human p = 0.20).

Confirming our hypothesis, results of one-way ANOVA revealed significant (p<0.05) mean intergroup differences for slow-twitch myosin percentages in the ZM and the OOM (see Table S2). Humans had the highest proportion of slow-twitch fibers in both muscles (about 15% in the ZM and 20% in the OOM). Chimpanzees (less than 1% in the ZM and 7% in the OOM) and rhesus macaques (about 5% in the ZM and 7% in the OOM) had far lower percentages of slow-twitch fibers in both muscles. These differences were statistically (p<0.05) significant for both the ZM (df = 2, p = 0.03) and the OOM (df = 2, p = 0.00). Results of *post-hoc* Least Squares Difference testing revealed that humans had the significantly highest percentage of slow-twitch fibers in the OOM relative to chimpanzees and macaques (human > chimpanzee = rhesus macaque; chimpanzee v. human p = 0.002; chimpanzee v. rhesus macaque p = 0.26; rhesus macaque v. human p = 0.000).

For the ZM, *post-hoc* Least Squares Difference testing revealed that humans shared the highest percentage of slow-twitch fibers with chimpanzees (chimpanzee v. human p = 0.21). However the unusually high standard error of the mean (SEM) in the human percentage of slow-twitch fibers here may have clouded statistical significance in the difference in mean percentages between humans and chimpanzees (human slow-twitch 15%, chimpanzee slow-twitch 8%) (see Figure 3). These *post-hoc* tests indicated that chimpanzees had no mean difference in percentage of slow-twitch fibers compared to rhesus macaques (chimpanzee v. rhesus macaque p = 0.49) but that humans had significantly greater mean percentage of slow-twitch fibers than rhesus macaques (rhesus macaque v. human p = 0.02).

These results show that chimpanzees and rhesus macaques tend to have greater percentages of fast-twitch myosin fibers in facial muscles while humans tend to have greater percentages of slow-twitch myosin fibers. While humans and chimpanzees did not statistically differ in percentage of slow-twitch myosin fibers in the zygomaticus major muscle, this may be due to the unusually high SEM in the human mean percentage.

Additionally, this may be due in part to the greater usage of the orbicularis oris muscle in speech than the zygomaticus major muscle. Humans use the orbicularis oris muscle for many functions, including shaping the lips as speech articulators [27]–[29] while chimpanzees use it in shaping the lips as a prehensile tool for grooming and vocalization purposes, such as the pant-hoot where the lips are strongly funneled [5], [30]–[32]. Previous histochemical microanatomical studies comparing chimpanzee and human orbicularis oris muscles have shown differences in muscle fiber organization and metrics [33], reflecting the divergence in usage of this muscle. The evolutionary divergence of lip function between humans and chimpanzees may be partially responsible for the greater percentage of slow-twitch fibers in the human orbicularis oris muscle. The zygomaticus major muscle is not reported to be an important speech articulator muscle in humans [10], [28] but is used in a range of human facial displays such as the spontaneous smile [21]. Chimpanzees use this muscle in a number of facial displays as well [5]. Thus, there may not be an evolutionary divergence in function between chimpanzees and humans in this muscle as found in the orbicularis oris muscle.

Rhesus macaques typically grouped with chimpanzees in percentages of fiber types except for the zygomaticus major, fast-twitch percentage, where they had a lower mean percentage than chimpanzees. Overall, there seems to be little differentiation between rhesus macaques and chimpanzees in fiber-type percentages, though. This may mirror the relative similarity in function of these muscles, producing a variety of facial displays that occur quickly and last only a short period of time [4].

Previous studies comparing the gross anatomical aspects of facial musculature among primate species failed to demonstrate derived aspects of human facial musculature [34]–[36]. Results of the present study suggest that the unique aspects of human facial musculature and facial expressions may be physiologic in nature, rather than anatomical. While minimal anatomical differences exist among the facial musculature of humans, chimpanzees, and rhesus macaques [35], [36], the present study revealed physiologic differences among slow-twitch myosin fiber proportions among these species.

These results also offer a new conceptual framework for our understanding of the evolution of human facial musculature. While humans continue to use facial muscles as a means of producing facial expressions of emotion and intent, a new selective pressure on these muscles may have been at work during human evolution: the development of speech. During speech, mimetic musculature generates “visemes”, visual phonemes. The evolutionary innovation of speech required sustained, deliberate control of the lips to form these visemes which favored the evolutionary novelty of a physiologic bias toward a higher percentage of slow-twitch myosin fibers in the human face.

## Supporting Information

Table S1 Mean (+ standard error of the mean) percentages of fast-twitch myosin fibers by study group and muscle. Note: The “raw” value represents the mean, untransformed percentage; the “transformed” value represents the mean percentage derived from the arc-sine transformed percentages. Statistical testing was conducted on transformed values. Note that results of one-way ANOVA testing revealed significant (p<0.05) differences among the three study groups in both muscles. Abbreviations: ZM – zygomaticus major muscle; OOM – orbicularis oris muscle.
(DOCX)

Table S2 Mean (+ standard error of the mean) percentages of slow-twitch myosin fibers by study group and muscle. Mean (± standard error of the mean) percentages of slow-twitch myosin fibers by study group and muscle.
(DOCX)
