# Supplementary material for: Human Faces Are Slower than Chimpanzee Faces
Source: PLoS One. 2014 Oct 22;9(10):e110523. doi: 10.1371/journal.pone.0110523 (PMC4206419; doi:10.1371/journal.pone.0110523)
Supplement: Table S1 — Mean (+ standard error of the mean) percentages of fast-twitch myosin fibers by study group and muscle. Note: The “raw” value represents the mean, untransformed percentage; the “transformed” value represents the mean percentage derived from the arc-sine transformed percentages. Statistical testing was conducted on transformed values. Note that results of one-way ANOVA testing revealed significant (p<0.05) differences among the three study groups in both muscles. Abbreviations: ZM – zygomaticus major muscle; OOM – orbicularis oris muscle. (DOCX) [file pone.0110523.s001.docx]

**Table S1** Mean (+ standard error of the mean) percentages of fast-twitch myosin fibers by study group and muscle

Group ZM OOM____________________

raw transformed raw transformed__________

Human 60.0 (7.9) 0.66 (0.07) p=0.03 90.90 (2.08) 1.28 (0.03) p=0.01

Rhesus macaque 80.50 (2.9) 0.80 (0.03) 93.0 (1.8) 1.33 (0.03)

Chimpanzee________97.10 (0.6)__________0.92 (0.01)______________ 95.60 (1.18)__1.37 (0.01)___________

Note: The “raw” value represents the mean, untransformed percentage; the “transformed” value represents the mean percentage derived from the arc-sine transformed percentages. Statistical testing was conducted on transformed values. Note that results of one-way ANOVA testing revealed significant (p<0.05) differences among the three study groups in both muscles. Abbreviations: ZM – zygomaticus major muscle; OOM – orbicularis oris muscle
