# Supplementary material for: Human Faces Are Slower than Chimpanzee Faces
Source: PLoS One. 2014 Oct 22;9(10):e110523. doi: 10.1371/journal.pone.0110523 (PMC4206419; doi:10.1371/journal.pone.0110523)
Supplement: Table S2 — Mean (+ standard error of the mean) percentages of slow-twitch myosin fibers by study group and muscle. Mean (± standard error of the mean) percentages of slow-twitch myosin fibers by study group and muscle. (DOCX) [file pone.0110523.s002.docx]

**Table S2** Mean (+ standard error of the mean) percentages of slow-twitch myosin fibers by study group and muscle

Group ZM OOM____________________

raw transformed raw transformed__________

Human 14.60 (5.92) 0.15 (0.06) p=0.03 20.40 (3.13) 0.20 (0.03) p = 0.01

Rhesus macaque 4.74 (0.92) 0.05 (0.01) 6.80 (1.5) 0.12 (0.01)

Chimpanzee_________0.93 (0.25) _______0.08 (0.02) _______________7.10 (1.55)_ 0.09 (0.01)____________

Note: The “raw” value represents the mean, untransformed percentage; the “transformed” value represents the mean percentage derived from the arc-sine transformed percentages. Statistical testing was conducted on transformed values. Note that results of one-way ANOVA testing revealed significant (p<0.05) differences among the three study groups in both muscles. Abbreviations: ZM – zygomaticus major muscle; OOM – orbicularis oris muscle
